# Regional Trends and Forecasts of Pancreatic Cancer Incidence in Poland: A Voivodeship-Level Analysis of Risk Factors

**DOI:** 10.3390/jcm15124724

**Published:** 2026-06-18

**Authors:** Sławomir Porada, Aleksandra Czerw, Natalia Czerw, Olga Partyka, Monika Pajewska, Tomasz Banaś, Izabela Gąska, Elżbieta Kaczmar, Katarzyna Sygit, Marian Sygit, Paulina Wojtyła-Buciora, Jarosław Drobnik, Piotr Pobrotyn, Dorota Waśko-Czopnik, Tomasz Sowiński, Katarzyna Tejza, Wojciech Homola, Łukasz Strzępek, Mateusz Curyło, Monika Urbaniak, Marcin Mikos, Elżbieta Grochans, Anna M. Cybulska, Daria Schneider-Matyka, Kamila Rachubińska, Ewa Bandurska, Weronika Ciećko, Monika Borzuchowska, Artur Budzyński, Remigiusz Kozlowski

**Affiliations:** 1 Faculty of Health Sciences and Psychology, Collegium Medicum, University of Rzeszów, 35-959 Rzeszow, Poland; 2 Department of Health Economics and Insurance, Medical University of Warsaw, 00-581 Warsaw, Poland; 3 Department of Epidemiology, Maria Sklodowska-Curie National Research Institute of Oncology, Krakow Branch, 31-115 Krakow, Poland; 4 Students’ Scientific Organization of Cancer Cell Biology, Department of Oncology Propaedeutics, Medical University of Warsaw, 01-445 Warsaw, Poland; 5 Department of Radiotherapy, Maria Sklodowska-Curie National Research Institute of Oncology, Krakow Branch, 31-115 Krakow, Poland; 6 Medical Institute, Jan Grodek State University in Sanok, 38-500 Sanok, Poland; 7 Faculty of Medicine and Health Sciences, University of Kalisz, 62-800 Kalisz, Poland; 8 Department of Family Medicine, Faculty of Medicine, Wroclaw Medical University, 50-368 Wroclaw, Poland; 9 Department of Clinical Neurosciences, Faculty of Medicine, Wroclaw University of Science and Technology, 50-370 Wroclaw, Poland; 10 Department of Gastroenterology, Hepatology with Inflammatory Bowel Disease Subunit, Provincial Specialist Hospital J. Gromkowskiego, 51-149 Wroclaw, Poland; 11 Endocare Medical Center, Simple Joint-Stock Company (S.J.S.C.), 50-558 Wroclaw, Poland; 12 FemiMea Centre for Obstetrics and Gynaecology, 55-040 Bielany Wroclawskie, Poland; 13 Clinical Department of General, and Oncological Surgery, Saint Raphael Hospital, 30-693 Krakow, Poland; 14 Department of Surgery, Andrzej Frycz Modrzewski Krakow University, 30-705 Krakow, Poland; 15 Institute of Health Sciences, University of the National Education Commission, 30-084 Krakow, Poland; 16 Rehabilitation Department, Hospital of the Ministry of Interior and Administration in Krakow, 02-507 Krakow, Poland; 17 Department of Medical and Pharmaceutical Law, Faculty of Medicine, University of Medical Sciences, 61-701 Poznan, Poland; 18 Department of Bioinformatics and Public Health, Andrzej Frycz Modrzewski Krakow University, 30-705 Krakow, Poland; 19 Department of Nursing, Faculty of Health Sciences, Pomeranian Medical University in Szczecin, 71-210 Szczecin, Poland; 20 Center for Competence Development, Integrated Care, and e-Health, Medical University of Gdansk, 80-204 Gdansk, Poland; 21 Department of Management, Faculty of Management, University of Lodz, 90-237 Lodz, Poland; 22 Department of Management and Logistics in Healthcare, Medical University of Lodz, 90-131 Lodz, Poland

**Keywords:** pancreatic, cancer, neoplasm, risk factors

## Abstract

**Background:** Pancreatic cancer is characterized by increasing incidence and high mortality in Poland and worldwide. The aim of this study was to assess the relationship between selected risk factors and the age-standardized incidence rate of pancreatic cancer at the voivodeship level in Poland, and to evaluate the accuracy of a prediction model. **Methods:** Age-standardized incidence rate data for 16 Polish voivodeships in 2011–2023 were obtained from the Polish National Cancer Registry. The risk factor burden for 2011–2019, expressed as disability-adjusted life years (DALYs) per 100,000 population, was obtained from the System Analysis and Implementation Database of the Polish Ministry of Health. A generalized estimating equation model was constructed to predict the age-standardized incidence rate, with multicollinearity addressed using variance inflation factor analysis. Predictions for 2020–2023 were validated against observed data, and forecasts for 2024–2030 were subsequently calculated. **Results:** The number of new pancreatic cancer cases in Poland increased in eight out of 16 voivodeships. The highest burden was recorded in the Masovian, Subcarpathian, Świętokrzyskie and Greater Poland voivodeships. Air pollution was positively associated with pancreatic cancer incidence. Predictions for 2020–2023 showed satisfactory agreement with observed data, with the largest discrepancy being equal to 4.1 in terms of the age-standardized incidence rate. Based on the models, the incidence of pancreatic cancer was projected for all of 16 voivodeships through to 2030. **Conclusions:** Air pollution is associated with the regional burden of pancreatic cancer in Poland. The generalized estimating equation prediction approach demonstrated acceptable accuracy and can support monitoring and public health planning at the voivodeship level.

## 1. Introduction

The World Health Organization reported the incidence of pancreatic cancer to be equal to 510,922 cases worldwide in 2022 [1]. The age-standardized incidence rate is equal to 4.7/100,000, which makes it the 12th most prevalent malignancy. The incidence rate values for Europe and North America were higher, equal to 8.0/100,000 and 8.5/100,000, respectively, and for Oceania, that value was 6.2/100,000 [1,2]. The highest incidence rates were recorded in Uruguay and Hungary, with the values of 11.4/100,000 and 10.4/100,000, respectively. In Poland, 5881 cases in 2022 and an age-standardized incidence rate equal to 6.7/100,000 were reported.

Regarding gender, the incidence rates were higher in males than in females [1,2]. In the total world population, they were equal to 5.5/100,000 and 4.0/100,000 for males and females, respectively. In North America, values of 9.6/100,000 and 7.4/100,000; in Oceania, values of 6.6/100,000 and 5.8/100,000; in Europe, values of 9.5/100,000 and 6.7/100,000; and in Asia, values of 4.3/100,000 and 3.0/100,000 were reported for males and females, respectively. In Poland, the incidence rate for males was equal to 8.1/100,00 and for females to 5.5/100,00. Therefore, in Europe, the incidence rates for males and females differed similarly in Poland and in Europe in general.

The reported risk of developing pancreatic cancer based on data from the European Commission by age 74 is 1/125 for women and 1/91 for men [2]. The likelihood of five-year survival is low and estimated to be 10% worldwide [3]. In some countries, i.e., the Netherlands, Denmark and Finland, the percentage is much lower. In Poland, it is also lower than 10% [2].

Regarding mortality, pancreatic cancer is sixth on the list of malignancy-related deaths. The global age-standardized mortality was equal to 4.2/100,000 [1]. Mortality rates were higher for North America at 6.6/100,000, Europe at 7.3/100,000, and Oceania at 5.4/100,000. In Poland, the age-standardized mortality rate in 2022 was equal to 6.5/100,00. The mortality rates were also higher in males compared with females. In North America, they were equal to 7.7/100,000 and 5.6/100,000, in Oceania to 6.2/100,000 and 4.6/100,000, in Europe to 8.8/100,000 and 5.9/100,000, and in Asia to 3.9/100,000 and 2.7/100,000 for males and females, respectively. In Poland, the age-standardized mortality rate in 2022 was equal to 7.8/100,00 for males and to 5.3/100,000 for females. Therefore, the differences between females and males were larger in Poland than in Europe overall. Over time, incidence rates increased, and mortality rates stayed high, which could be attributed to the lack of an effective screening method and late-stage diagnoses as a result [3].

The main risk factors for pancreatic cancer are metabolic factors. These include type 2 diabetes mellitus (T2DM) [4], which is associated with a 1.5 to 2.0 times increase in the risk. However, the causal role of T2DM may be confounded by related metabolic factors, i.e., hyperinsulinemia and obesity [5]. Also, the distinction between long-standing diabetes and new-onset diabetes is important, as diabetes diagnosed within one year increases the risk of pancreatic cancer sevenfold, while new-onset diabetes is associated with a risk that is three times higher relative to the general population, and long-standing diabetes doubles the risk of pancreatic cancer [6]. However, the elevated risk in new-onset diabetes can be attributed to reverse causality, as pancreatic cancer itself can induce diabetes [7]. Insulin resistance, which is associated with hyperglycemia, hyperinsulinemia, and inflammation, may be the underlying mechanism contributing to the development of diabetes-associated pancreatic cancer [8]. Type 3c diabetes, which is a consequence of exocrine pancreatic diseases, including chronic pancreatitis and pancreatic ductal adenocarcinoma, is also an important risk factor [9].

Another well-established risk factor for pancreatic cancer is obesity [10]. The proportion of disability-adjusted life years due to pancreatic cancer attributable to high BMI has increased in the perspective of the last three decades. Pancreatic cancer is categorized as one of the 13 obesity-related cancers. Obesity is also linked to metabolic syndrome, which is associated with pancreatic cancer risk as well [5].

Elevated glycemic markers were found to be independently associated with pancreatic cancer risk. Higher HbA1c and higher fasting or random glucose have been associated with higher pancreatic cancer risk independent of BMI, even in the non-diabetic range [10]. Acute and chronic pancreatitis is also a significant risk factor for pancreatic cancer [11]. The combination of T2DM and acute pancreatitis, including alcohol acute pancreatitis, is associated with an elevated risk. Also, cholecystitis and cholelithiasis, both independently and when combined with T2DM, are associated with an increased risk of pancreatic cancer. Alcohol dependence and alcoholic liver disease have been highlighted as risk factors for pancreatic cancer as well. Gastric ulcer is also associated with an increased risk of pancreatic cancer in combination with T2DM.

Smoking is also a risk factor for pancreatic cancer and for T2DM as well. It is estimated that 20–30% of all pancreatic cancer cases are attributable to cigarette smoking [12].

The impact of risk factors can be measured in terms of disability-adjusted life years (DALYs), which is a measure combining both premature death and disability resulting from a disease [13]. It has components: the number of deaths multiplied by the standard life expectancy at the age of death and the number of prevalent cases multiplied by a disability weight, which ranges from 0 to 1. One DALY equals one lost year of a healthy life.

The age-standardized incidence rate determines how many cases of disease would occur in the studied population if the age structure of that population were the same as the age structure of the population adopted as the standard. The standard in the current study was the European Standard Population (ASE).

The purpose of the current paper is to assess the relationship between the estimates of risk factors and the estimates of the age-standardized incidence rate of pancreatic cancer on the basis of the data on the level of Polish voivodeships (regional governmental units) and to evaluate the accuracy of a prediction model. To our best knowledge, no such approach was published so far. It combines official reliable data regarding risk factors and cancer incidence for making predictions.

## 2. Materials and Methods

Poland is administratively divided into 16 voivodeships. The estimates of the age-standardized incidence rate of pancreatic cancer in each of the 16 voivodeships in 2011–2023 were acquired from the Polish National Cancer Registry [14]. It is a population-based registry, collecting data on new cancer cases in Poland. The range of collected data follows the guidelines of international organizations of cancer registries that are operating under the World Health Organization and the European Commission. It allows for studying cancer epidemiology at the national and voivodeship levels.

The share of risk factors in 2011–2019 in Poland was acquired from the System Analysis and Implementation Database provided by the Polish Ministry of Health [15]. The system allows for acquiring the estimates regarding risk factors expressed as DALYs per 100,000 people. They represent the total burden attributable to each risk factor across all diseases.

The acquired data regarding risk factors in 2011–2019, including metabolic risk factors, low physical activity, alcohol consumption, smoking, high BMI, air pollution and water pollution, were included in the generalized estimating equation model [16] as predictors of the age-standardized incidence rate of pancreatic cancer in 2011–2019.

Multicollinearity was assessed with variance inflation factor values, and three regression analysis models were calculated to avoid including strongly correlated predictors in a single statistical model. Next, the predictions based on the generalized estimating equation model regarding the years 2020–2023 were verified by comparison with the actual data regarding the age-standardized incidence rate of pancreatic cancer in this period. Finally, the predictions for the years 2024–2030 were calculated. A voivodeship in a specific year was the unit for the analyses performed.

The calculations were performed with the use of the IBM SPSS Statistics 31.0 software.

## 3. Results

Table 1 depicts the age-standardized incidence rate of pancreatic cancer for each voivodeship in 2011–2023 according to the Polish National Cancer Registry.

The age-standardized incidence rate of pancreatic cancer increased in the Kuyavian–Pomeranian, Lublin, Lodz, Masovian, Opole, Podlaskie, Greater Poland and West Pomeranian voivodeships, i.e., in eight out of 16 voivodeships. The highest incidence rates were detected in the Masovian, Subcarpathian, Świętokrzyskie and Greater Poland voivodeships.

The burden of risk factors included in the analysis in 2011–2019 expressed as DALYs per 100,000 people according to the System Analysis and Implementation Database provided by the Polish Ministry of Health is provided in Table 2. The table lists the average, minimum and maximum values for DALYs regarding the risk factors for each voivodeship. The values for DALYs were established empirically on the basis of the Global Burden of Disease data acquired from the Institute for Health Metrics and Evaluation regarding the period of 1991–2023. It is the only comprehensive database providing estimates of risk factors for each voivodeship in Poland.

The highest DALYs were attributed to metabolic risk factors, and the lowest to water pollution. The highest levels of risk factors were detected in the Lublin and Silesian voivodeships, and the lowest in the Subcarpathian and Pomeranian voivodeships.

The data regarding risk factors and the incidence of pancreatic cancer were analyzed further with multiple linear regression analysis. Firstly, multicollinearity was assessed by examining variance inflation factor values. The VIF values were equal to 22.62, 12.71, 21.22, 2.84, 2.84, 4.52 and 4.57 for metabolic risk factors, low physical activity, high BMI, alcohol consumption, smoking, air pollution and water pollution, respectively.

The values for three predictors, i.e., metabolic risk factors, low physical activity and high BMI, exceeded the value of 10, indicating serious multicollinearity, which could inflate the variance in the coefficient estimates and lead to unreliable statistical inferences [17,18]. Collinearity diagnostics revealed that all three were highly correlated with each other. The proportion of variance for the same dimension was equal to 0.73 for metabolic risk factors and to 0.83 for high BMI. In another dimension, the proportion of variance was equal to 0.26 for metabolic risk factors and to 0.85 for low physical activity. To avoid unreliable statistical inferences, the three predictors were subjected to principal component analysis. The extracted common component was used as one of the predictors in further analysis. The factor loadings were equal to 0.98, 0.97 and 0.96 for high BMI, low physical activity and metabolic risk factors, respectively. The component accounted for 93.7% of the variance.

The data regarding risk factors and the age-standardized incidence rate of pancreatic cancer were analyzed further with generalized estimating equation modeling. The results are depicted in Table 3.

The only statistically significant predictor was air pollution. It was related positively to the incidence of pancreatic cancer, which means that the voivodeships with a higher burden of air pollution exhibited a higher age-standardized incidence rate.

For the purpose of prediction, all predictors were used, including the ones for which statistical significance was not detected. This solution was chosen for two reasons: firstly, to maintain the values of determination coefficients and the accuracy of predictions as high as possible; secondly, the calculations were based on comprehensive data with all voivodeships included so that the data analyzed were not a sample based on some voivodeships, which makes statistical significance, allowing for extending conclusions based on a sample to the population, less relevant.

On the basis of the generalized estimating equation model, predictions regarding the age-standardized incidence rate of pancreatic cancer were calculated.

Also, the predictions for 2020–2023 were compared with the actual data available. For the purpose of evaluating predictions, the values of risk factors and DALY estimators were forecasted with the use of the linear trend. Table A1 in Appendix A depicts the estimated incidence values.

**Figure 1:**
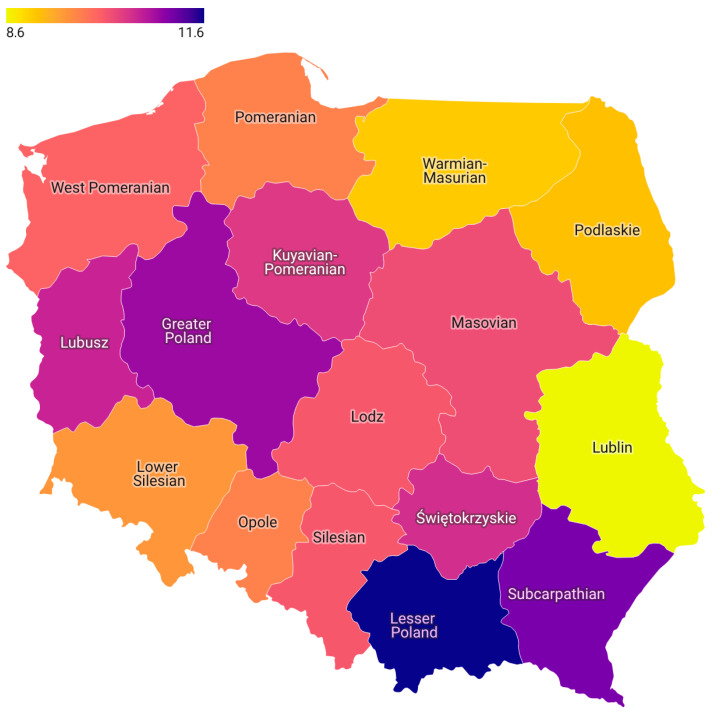
Map of Poland with a color gradient to show the predicted provincial incidence of pancreatic cancer in 2030 based on the generalized estimating equation model.

Figure 1 depicts a map of Poland with a color gradient to show the predicted provincial incidence of pancreatic cancer in 2030.

**Figure 2:**
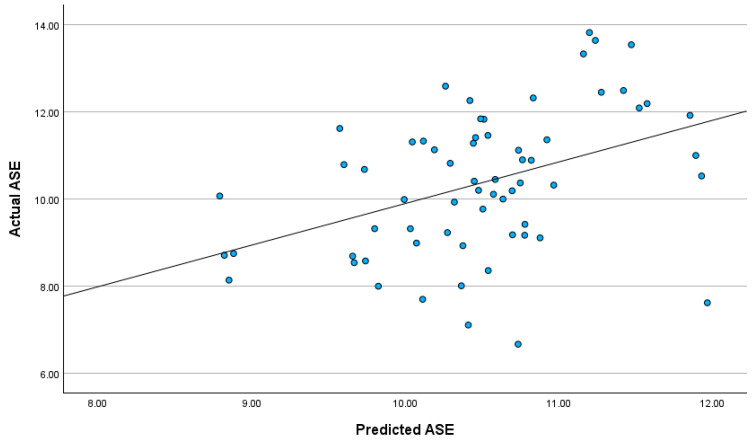
The relationship between the predicted values and the actual data for 2020–2023 based on the generalized estimating equation model.

The differences between predictions and the actual data available for 2020–2023 were highest for the Lublin, Masovian and Greater Poland voivodeships (underestimation) and for the Lubusz voivodeship (overestimation). Figure 2 depicts the relationship between the predicted values and the actual data for 2020–2023.

## 4. Discussion

The age-standardized incidence rate of pancreatic cancer increased in the Kuyavian–Pomeranian, Lublin, Lodz, Masovian, Opole, Podlaskie, Greater Poland and West Pomeranian voivodeships, i.e., in eight out of 16 voivodeships. The predictions were based on seven risk factors, specifically, metabolic risk factors, low physical activity, alcohol consumption, smoking, high BMI, air pollution and water pollution. However, metabolic risk factors, high BMI and low physical activity needed to be analyzed as a single component due to strong associations between these three risk factors.

Metabolic risk factors, high BMI and low physical activity as a single factor were not related to the incidence of pancreatic cancer. This finding is inconsistent with the current state of knowledge [5,10]. Obesity, diabetes, and lack of physical activity are well-established risk factors for pancreatic cancer [19,20,21]. Also, the observed rising incidence of pancreatic cancer among younger adults is hypothesized to be associated, partly, with increasing rates of obesity and metabolic dysfunction at younger ages [22].

Alcohol consumption was not related to the incidence of pancreatic cancer in the current study, which is inconsistent with other scientific papers on the subject [11]. A large-scale pooled analysis [23] revealed a positive association between alcohol intake and pancreatic cancer risk, when controlling for sex and smoking status. Specifically, intake exceeding the threshold values equal to 15 g/day in females and 30 g/day in males was found to be evidently associated. A systematic review based on 80 cohort and case–control studies [24] concluded that heavy alcohol consumption, i.e., at least three drinks a day, was consistently associated with an increased risk of pancreatic cancer, particularly in males.

The result of the current study revealing the positive association between air pollution and the incidence of pancreatic cancer is consistent with the results of other research projects. A multiethnic cohort study [25] identified an association between fine particulate matter, PM2.5, and pancreatic cancer on 1,660,488 person-years accumulated over the period of the study. The average follow-up time was over 16 years. Another study [26] including 203 cases of pancreatic cancer and 5027 controls revealed that patients with pancreatic cancer had higher average exposure to PM2.5 annually, which was associated with a greater risk of pancreatic cancer.

However, statistical models provide estimates for individual predictors taking other predictors included in the analysis into account. As a consequence, a conclusion for each predictor should be made with awareness that it is only valid when controlling for other predictors included in the model. With a different set of predictors, conclusions may be different due to the relationships between predictors included and the strength of the relationships between the predictors and the outcome analyzed. In the current study, metabolic risk factors were related positively with alcohol intake (r = 0.608; *p* < 0.001), smoking (r = 0.564; *p* < 0.001) and air pollution (r = 0.799; *p* < 0.001). Also, high BMI was related positively with alcohol intake (r = 0.596; *p* < 0.001), smoking (r = 0.659; *p* < 0.001) and air pollution (r = 0.729; *p* < 0.001). Finally, low physical activity was related positively with alcohol intake (r = 0.418; *p* < 0.001), smoking (r = 0.401; *p* < 0.001) and air pollution (r = 0.883; *p* < 0.001).

The main limitation of the current paper is that the risk factor that is widely recognized and modifiable, i.e., type 2 diabetes mellitus, was not included in the analysis. Unfortunately, the data regarding the prevalence of T2DM in Poland for each of the 16 voivodeships are available only for every 10 years, starting with 1999 and ending with 2019 [27]. The years 1999 and 2009 are beyond the scope of our analysis. The correlation between the prevalence of T2DM and the age-standardized incidence rate of pancreatic cancer for 2019 was not statistically significant (r = −0.182; *p* = 0.500). However, it was calculated including only 16 data points. A similar analysis performed on global data with diabetes type 2 incidence included could yield interesting results [28]. However, elevated BMI and metabolic risk factors in general were included in the analysis, and they both indirectly capture either being at higher risk of developing or suffering from T2DM. The analysis and the conclusions would certainly benefit from adding more clinical and biological data, if they are available in the future.

Another limitation is that the conclusions from the analysis based on large data units like voivodeships cannot be easily transferred to individuals. However, on the level relevant to the perspective of public health management, like the level of voivodeships, the analysis depicted in the current paper shows that the incidence of pancreatic cancer will remain serious in the coming years. Also, a policy regarding air pollution, if effective, can change the expected trend.

## 5. Conclusions

Pancreatic cancer will remain a substantial health challenge in Poland. The observed association between pancreatic cancer incidence and the burden of air pollution risk factor expressed in DALYs underscores the importance of this determinant for the current and future epidemiological situation.

Using routinely collected registry data together with DALY-based estimates of risk factors allowed for the development of generalized estimating equation models that reproduced recent trends and generated short-term forecasts up to 2030. These findings indicate that such an approach can support monitoring of pancreatic cancer burden at the voivodeship level and provide quantitative estimates that may be useful for planning and evaluating public health activities in Poland.

## Figures and Tables

**Table 1:** Age-standardized incidence rate for each voivodeship.

	Voivodeship															
Year	1.	2.	3.	4.	5.	6.	7.	8.	9.	10.	11.	12.	13.	14.	15.	16.
2011	11.61	9.08	8.37	9.50	10.79	11.99	8.44	9.91	13.09	9.45	10.39	12.02	12.70	12.75	12.59	7.98
2012	12.90	10.83	9.92	13.09	11.86	12.26	8.62	12.92	11.49	8.87	14.54	12.00	11.54	10.55	15.11	7.18
2013	12.32	10.85	11.20	8.14	10.98	12.39	8.35	11.71	13.50	8.78	11.57	11.20	11.08	9.54	14.10	11.04
2014	12.81	13.83	9.69	8.81	10.86	12.05	9.03	12.24	13.22	8.60	11.85	11.03	10.42	9.48	12.80	11.59
2015	13.60	11.16	8.54	9.87	9.80	10.98	9.89	10.16	14.19	9.83	13.82	9.97	9.20	13.26	12.68	9.84
2016	13.07	12.06	8.80	10.41	8.77	9.33	9.24	10.80	12.56	6.89	14.53	10.04	9.61	9.40	12.98	8.81
2017	11.26	12.35	10.48	9.64	8.47	9.02	8.52	11.70	10.85	6.88	13.46	11.05	12.25	10.41	14.50	6.16
2018	11.45	12.47	9.08	7.87	7.95	9.57	11.39	10.08	14.30	8.98	12.55	12.42	10.49	9.95	15.24	7.54
2019	13.58	13.45	9.17	11.65	10.33	9.12	9.95	8.97	14.54	8.36	11.41	10.41	11.45	9.26	13.47	7.53
2020	12.59	10.89	8.75	9.17	9.18	7.62	9.77	8.36	12.19	9.32	7.70	10.45	10.32	8.00	12.45	7.11
2021	11.13	9.42	8.14	10.90	10.00	10.53	10.20	11.41	12.09	10.68	8.99	11.46	11.36	8.58	13.64	8.01
2022	11.33	11.12	8.71	10.37	10.11	11.00	10.41	8.93	13.54	8.54	9.32	11.84	9.11	8.69	13.82	9.93
2023	11.31	10.19	10.07	6.67	11.83	11.92	12.26	10.82	12.49	10.79	9.99	11.28	12.32	11.62	13.33	9.23

1. Lower Silesia; 2. Kuyavian–Pomeranian; 3. Lublin; 4. Lubusz; 5. Lodz; 6. Lesser Poland; 7. Masovian; 8. Opole; 9. Subcarpathian; 10. Podlaskie; 11. Pomeranian; 12. Silesian; 13. Świętokrzyskie; 14. Warmian–Masurian; 15. Greater Poland; 16. West Pomeranian. Source. Polish National Cancer Registry.

**Table 2:** The share of risk factors in 2011–2019 in Poland.

Voivodeship	1.	2.	3.	4.	5.	6.	7.
Lower Silesia	9247 [9009; 9551]	395 [380; 416]	2847 [2817; 2895]	6242 [6122; 6342]	4229 [4089; 4436]	1972 [1860; 2200]	40 [37; 41]
Kuyavian–Pomeranian	8266 [7985; 8635]	321 [309; 342]	2537 [2515; 2579]	6140 [5990; 6322]	3676 [3549; 3903]	1630 [1513; 1853]	43 [40; 45]
Lublin	10,422 [10,099; 10,694]	409 [397; 426]	3643 [3552; 3768]	6659 [6477; 6847]	4446 [4306; 4621]	2465 [2354; 2774]	56 [53; 58]
Lubusz	8882 [8663; 9179]	358 [352; 371]	2642 [2585; 2774]	5495 [5379; 5692]	3759 [3671; 3929]	1809 [1703; 2071]	45 [41; 47]
Lodz	8771 [8502; 9115]	333 [322; 352]	2725 [2679; 2774]	5929 [5767; 6089]	3949 [3822; 4167]	1775 [1692; 2007]	39 [36; 41]
Lesser Poland	8027 [7828; 8201]	347 [338; 361]	2088 [2035; 2131]	4870 [4739; 5027]	3462 [3372; 3610]	1980 [1873; 2172]	47 [44; 49]
Masovian	8644 [8429; 8785]	358 [348; 372]	2814 [2753; 2921]	5336 [5198; 5511]	3917 [3815; 4050]	1779 [1687; 2018]	44 [41; 46]
Opole	9811 [9413; 10,307]	393 [373; 421]	2397 [2330; 2425]	5773 [5528; 5970]	4300 [4128; 4579]	2122 [2031; 2310]	47 [44; 49]
Subcarpathian	7576 [7256; 7906]	303 [288; 322]	2125 [2088; 2161]	4366 [4207; 4510]	3240 [3100; 3469]	1574 [1505; 1717]	48 [44; 50]
Podlaskie	8337 [8107; 8652]	313 [295; 330]	2941 [2883; 2969]	4786 [4635; 4909]	3501 [3323; 3733]	1449 [1381; 1597]	49 [46; 51]
Pomeranian	7779 [7535; 8083]	298 [279; 315]	2520 [2481; 2591]	5247 [5147; 5347]	3457 [3255; 3651]	1195 [1141; 1327]	51 [47; 53]
Silesian	10,169 [9907; 10,541]	405 [379; 432]	3100 [3028; 3209]	5786 [5661; 5930]	4419 [4252; 4635]	2488 [2373; 2675]	41 [37; 42]
Świętokrzyskie	9547 [9300; 9840]	388 [376; 409]	2623 [2572; 2683]	5880 [5727; 5963]	3997 [3880; 4201]	2074 [1984; 2296]	40 [37; 42]
Warmian–Masurian	8219 [7876; 8698]	311 [284; 336]	2928 [2862; 2966]	5882 [5623; 6128]	3617 [3389; 3902]	1492 [1403; 1659]	46 [43; 48]
Greater Poland	8122 [7901; 8386]	323 [309; 340]	2475 [2445; 2506]	5450 [5313; 5539]	3681 [3567; 3873]	1674 [1601; 1857]	40 [37; 42]
West Pomeranian	8477 [8170; 8829]	324 [312; 343]	2666 [2587; 2748]	6327 [6105; 6495]	3810 [3675; 4026]	1525 [1445; 1732]	42 [39; 43]

1. Metabolic risk factors; 2. low physical activity; 3. alcohol consumption; 4. smoking; 5. high BMI; 6. air pollution; 7. water pollution. Note. The table lists the average, minimum and maximum values in 2011–2019 for DALYs regarding the risk factors for each voivodeship. Source. Calculations based on System Analysis and Implementation Database provided by the Polish Ministry of Health.

**Table 3:** Results of generalized estimating equation analysis predicting age-standardized incidence of pancreatic cancer in a voivodeship on the basis of risk factors.

Predictors	*Exp*(*B*)	*p*
(Constant)	1,557,714.33 [5383.71; 450,706,398.82]	0.001
Metabolic risk factors	0.60 [0.33; 1.08]	0.089
High BMI		
Low physical activity		
Alcohol consumption	1.01 [0.95; 1.05]	0.121
Smoking	1.00 [0.98; 1.02]	0.864
Air pollution	1.02 [1.01; 1.03]	0.020
Water pollution	0.95 [0.85; 1.06]	0.332

*Exp*(*B*)—odds ratio with 95% confidence interval; *p*—statistical significance. Source. Calculations based on System Analysis and Implementation Database provided by the Polish Ministry of Health, Polish National Cancer Registry and Thematic Emission Monitoring Integrated Services.

## Data Availability

The data are contained within this article.

## Appendix A

**Table A1:** Predictions regarding incidence of pancreatic cancer for 2020–2030 rounded to integers with 95% confidence intervals.

	Voivodeship															
Year	1.	2.	3.	4.	5.	6.	7.	8.	9.	10.	11.	12.	13.	14.	15.	16.
Actual data																
2020	12.59	10.89	8.75	9.17	9.18	7.62	9.77	8.36	12.19	9.32	7.70	10.45	10.32	8.00	12.45	7.11
2021	11.13	9.42	8.14	10.90	10.00	10.53	10.20	11.41	12.09	10.68	8.99	11.46	11.36	8.58	13.64	8.01
2022	11.33	11.12	8.71	10.37	10.11	11.00	10.41	8.93	13.54	8.54	9.32	11.84	9.11	8.69	13.82	9.93
2023	11.31	10.19	10.07	6.67	11.83	11.92	12.26	10.82	12.49	10.79	9.99	11.28	12.32	11.62	13.33	9.23
Predictions																
2020	10.26 [9.26; 11.26]	10.82 [9.46; 12.18]	8.88 [7.72; 10.05]	10.78 [10.15; 11.41]	10.70 [9.51; 11.89]	11.97 [10.67; 13.27]	10.51 [9.72; 11.29]	10.54 [9.55; 11.53]	11.57 [10.25; 12.9]	9.80 [8.47; 11.13]	10.11 [8.9; 11.33]	10.59 [9.37; 11.8]	10.97 [10.03; 11.9]	9.83 [8.72; 10.93]	11.28 [10.21; 12.35]	10.41 [8.87; 11.95]
2021	10.19 [9.17; 11.21]	10.78 [9.39; 12.16]	8.85 [7.67; 10.03]	10.76 [10.11; 11.41]	10.64 [9.41; 11.86]	11.93 [10.65; 13.21]	10.48 [9.68; 11.28]	10.46 [9.45; 11.46]	11.52 [10.24; 12.81]	9.73 [8.41; 11.06]	10.07 [8.87; 11.28]	10.54 [9.31; 11.76]	10.92 [9.97; 11.88]	9.74 [8.61; 10.88]	11.24 [10.16; 12.32]	10.37 [8.79; 11.95]
2022	10.12 [9.06; 11.17]	10.74 [9.33; 12.15]	8.82 [7.63; 10.02]	10.75 [10.08; 11.42]	10.57 [9.31; 11.84]	11.89 [10.63; 13.15]	10.45 [9.64; 11.26]	10.38 [9.35; 11.4]	11.47 [10.22; 12.72]	9.67 [8.34; 11]	10.03 [8.84; 11.23]	10.49 [9.25; 11.73]	10.88 [9.9; 11.86]	9.66 [8.49; 10.83]	11.2 [10.11; 12.29]	10.32 [8.7; 11.94]
2023	10.05 [8.96; 11.13]	10.7 [9.26; 12.14]	8.79 [7.58; 10]	10.73 [10.04; 11.43]	10.51 [9.21; 11.81]	11.85 [10.61; 13.1]	10.42 [9.59; 11.25]	10.29 [9.24; 11.34]	11.42 [10.21; 12.63]	9.60 [8.27; 10.94]	9.99 [8.8; 11.18]	10.44 [9.19; 11.7]	10.83 [9.83; 11.84]	9.57 [8.36; 10.78]	11.16 [10.06; 12.26]	10.27 [8.61; 11.94]
2024	9.97 [8.86; 11.09]	10.65 [9.18; 12.12]	8.76 [7.53; 9.99]	10.72 [10; 11.44]	10.45 [9.11; 11.79]	11.82 [10.59; 13.05]	10.39 [9.55; 11.24]	10.21 [9.13; 11.29]	11.37 [10.19; 12.55]	9.53 [8.19; 10.88]	9.95 [8.77; 11.14]	10.4 [9.12; 11.67]	10.79 [9.76; 11.82]	9.49 [8.24; 10.75]	11.12 [10.01; 12.23]	10.23 [8.52; 11.94]
2025	9.9 [8.75; 11.06]	10.61 [9.11; 12.11]	8.73 [7.48; 9.98]	10.71 [9.96; 11.46]	10.39 [9; 11.77]	11.78 [10.56; 13]	10.36 [9.5; 11.23]	10.13 [9.02; 11.24]	11.32 [10.17; 12.47]	9.47 [8.11; 10.82]	9.91 [8.73; 11.1]	10.35 [9.06; 11.64]	10.74 [9.68; 11.81]	9.41 [8.1; 10.71]	11.08 [9.96; 12.21]	10.18 [8.43; 11.93]
2026	9.83 [8.64; 11.02]	10.57 [9.04; 12.11]	8.7 [7.43; 9.97]	10.69 [9.91; 11.47]	10.33 [8.9; 11.76]	11.74 [10.53; 12.95]	10.34 [9.45; 11.22]	10.05 [8.9; 11.19]	11.27 [10.14; 12.39]	9.4 [8.03; 10.77]	9.87 [8.69; 11.06]	10.3 [8.99; 11.62]	10.7 [9.6; 11.8]	9.32 [7.97; 10.68]	11.04 [9.9; 12.18]	10.14 [8.34; 11.94]
2027	9.76 [8.52; 10.99]	10.53 [8.96; 12.1]	8.67 [7.38; 9.97]	10.68 [9.87; 11.49]	10.26 [8.79; 11.74]	11.7 [10.51; 12.9]	10.31 [9.4; 11.21]	9.96 [8.78; 11.15]	11.22 [10.11; 12.32]	9.33 [7.95; 10.72]	9.83 [8.65; 11.02]	10.25 [8.91; 11.59]	10.66 [9.53; 11.78]	9.24 [7.83; 10.65]	11.01 [9.85; 12.16]	10.09 [8.25; 11.94]
2028	9.69 [8.41; 10.96]	10.49 [8.88; 12.09]	8.64 [7.32; 9.96]	10.66 [9.82; 11.51]	10.2 [8.68; 11.73]	11.67 [10.47; 12.86]	10.28 [9.35; 11.21]	9.88 [8.66; 11.1]	11.16 [10.08; 12.25]	9.27 [7.87; 10.67]	9.79 [8.6; 10.98]	10.21 [8.84; 11.58]	10.61 [9.45; 11.78]	9.16 [7.69; 10.62]	10.97 [9.79; 12.15]	10.05 [8.16; 11.94]
2029	9.61 [8.3; 10.93]	10.45 [8.8; 12.09]	8.61 [7.27; 9.96]	10.65 [9.77; 11.53]	10.14 [8.56; 11.72]	11.63 [10.44; 12.82]	10.25 [9.3; 11.2]	9.8 [8.53; 11.06]	11.11 [10.04; 12.18]	9.2 [7.78; 10.62]	9.75 [8.56; 10.95]	10.16 [8.76; 11.56]	10.57 [9.37; 11.77]	9.07 [7.54; 10.6]	10.93 [9.73; 12.13]	10 [8.06; 11.94]
2030	9.54 [8.18; 10.9]	10.41 [8.72; 12.09]	8.58 [7.21; 9.95]	10.63 [9.72; 11.55]	10.08 [8.45; 11.71]	11.59 [10.4; 12.78]	10.22 [9.25; 11.19]	9.72 [8.4; 11.03]	11.06 [10; 12.12]	9.13 [7.69; 10.57]	9.71 [8.51; 10.92]	10.11 [8.68; 11.54]	10.52 [9.28; 11.76]	8.99 [7.4; 10.58]	10.89 [9.67; 12.11]	9.96 [7.97; 11.94]

1. Lower Silesia; 2. Kuyavian–Pomeranian; 3. Lublin; 4. Lubusz; 5. Lodz; 6. Lesser Poland; 7. Masovian; 8. Opole; 9. Subcarpathian; 10. Podlaskie; 11. Pomeranian; 12. Silesian; 13. Świętokrzyskie; 14. Warmian–Masurian; 15. Greater Poland; 16. West Pomeranian. Source. Calculations based on System Analysis and Implementation Database provided by the Polish Ministry of Health and Polish National Cancer Registry.
